# Clinical study of chlamydia pneumoniae infection in patients with coronary heart disease

**DOI:** 10.1186/s12872-019-1099-y

**Published:** 2019-05-14

**Authors:** Lei Xue, Yan-Hong Liang, Yuan-Yuan Gao, Xiao-Juan Wang

**Affiliations:** General Department, Beijing Chaoyang Hospital, Capital Medical University, No. 8 Gong-Ti South Road, Beijing, 100020 China

**Keywords:** Chlamydia pneumoniae, Infection, Inflammatory factor, Coronary heart disease, Prognosis

## Abstract

**Background:**

This study aims to investigate the chlamydia pneumoniae infection (PC) in patients with coronary heart disease.

**Methods:**

A total of 92 patients with coronary heart disease, who were treated with percutaneous coronary intervention (PCI), were selected as the case group. In addition, 50 healthy people were enrolled as the control group. The incidences of CP infection and serum Chlamydia pneumoniae IgA antibody (CP-IgA), high sensitive C-reactive protein (hs-CRP), and interleukin-6 (IL-6) were compared in these two groups. The classification of coronary artery lesion, the incidence of perioperative cardiovascular events, and adverse prognosis events within six months after procedure were compared.

**Results:**

The incidence of CP infection in the case group was higher (42.4% vs. 0%, *P* < 0.05). Furthermore, 17 patients were at grade I, 39 patients were at grade II, and 36 patients were at grade III. The incidences for these three kinds of patients were 17.6, 30.8, and 66.7%. The incidence of CP infection at grade III was higher than that of grade I or II (*P* < 0.05). Serum CP-IgA, hs-CRP and IL-6 levels increased with the severity of the coronary artery disease (*P* < 0.05), and the serum hs-CRP and IL-6 levels of patients with perioperative cardiovascular events were higher (*P* < 0.05). Moreover, the serum CP-IgA levels of the patients with adverse prognosis events were also higher (*P* < 0.05).

**Conclusions:**

Patients with coronary heart disease have a high CP infection rate. The degree of infection is relevant to the severity of the coronary artery lesions and postoperative prognosis of patients, suggesting that CP infection may be an important factor affecting the incidence and prognosis of coronary heart disease.

## Background

Coronary heart disease (CHD) is the most important vascular disease that threatens human health and is one of the main diseases that cause disability and death in elderly patients [1, 2]. According to relevant statistics of the United States, in fatal cases of over 65 years old, more than 50% of patients die from CHD [3, 4]. Recently, percutaneous coronary intervention (PCI) has gradually become the main treatment for patients with CHD [5]. However, adverse prognostic events still occur in some patients with CHD after PCI [6, 7]. A recent study confirmed that [8] immunoinflammatory damage caused by pathogenic microorganism infection to the vascular wall plays an important role in atherosclerotic diseases. Chlamydia pneumoniae (CP) is a Chlamydia discovered in the 1980s. The results of related studies have revealed that CP has a high infection rate worldwide, and approximately 50% of adults and 10–20% of children are positive for serum CP antibodies [9]. CP infection can cause respiratory tract infections, such as pneumonia, bronchitis, sinusitis and pharyngitis, and is also correlated to CHD, endocarditis, meningitis, erythema nodosum, bronchial asthma and other diseases [10, 11]. Although researchers have conducted a number of studies on the correlation between CP infection and CHD in recent years, none of these studies investigated the correlation between CP infection and the prognosis of patients with CHD. Based on this, in the present study, the correlation between CP infection and the prognosis of patients with CHD was analyzed by investigating their current status and follow-up, providing an objective basis for clinical diagnosis and treatment.

## Methods

### Clinical data

A total of 92 patients with CHD, who underwent PCI in the General Department of Beijing Chaoyang Hospital from June 2016 to June 2017, were enrolled into the present study. These patients were assigned as the case group. All patients were admitted and underwent PCI for angina pectoris. Among these patients, 17 patients were stable angina pectoris and 75 patients were unstable angina pectoris. The exclusion criteria: patients with other heart diseases; patients with acute myocardial infarction history within three months before inclusion into the present study; patients with stroke, malignancy, infectious disease and autoimmune disease; patients with hepatorenal insufficiency. In addition, 50 healthy subjects, who received physical examination in the same period, were enrolled into the present study. These subjects were assigned as the control group and all of them were excluded from CHD by clinical examination. The differences in age, gender composition, smoking history and chronic diseases between these two groups were not statistically significant (*P* > 0.05, Table 1). The identification of chlamydia was after percutaneous intervention.

**Table 1:** The clinical characteristics of the patients in the two groups

Clinical data		case group(*n* = 92)		control group(*n* = 50)		
		Number of cases	constituent ratio	number of cases	constituent ratio	P
Gender	Male	78	84.8	41	82.0	0.58
	Female	14	15.2	9	18.0	1.23
Age	≥60	42	45.7	22	44.0	0.73
	<60	50	54.3	28	56.0	0.65
Smoking history	Yes	35	38.0	19	38.0	0.92
	No	57	62.0	31	62.0	0.71
Hypertension	Yes	34	37.0	18	36.0	0.83
	No	58	63.0	32	64.0	0.66
Diabetes	Yes	14	15.2	8	16.0	1.42
	No	78	84.8	42	84.0	0.47
Hyperlipidemia	Yes	16	17.4	10	20.0	1.16
	No	76	82.6	40	80.0	0.62

### Therapeutic methods

All patients in the case group underwent coronary angiography by digital subtraction angiography (DSA). PCI was performed for coronary arteries with a diameter of ≥2.0 mm and a severity of stenosis of ≥70%.

### Observation indicators and detection methods

On the next day, the subjects were included into the present study. On the day before the PCI, fasting peripheral venous blood samples were collected from these two groups of subjects, centrifuged to separate the serum at room temperature, and the serum was placed in a refrigerator at − 80 °C for detection. The levels of Chlamydia pneumoniae IgA antibody (CP-IgA), hypersensitive C-reactive protein (hs-CRP) and interleukin-6 (IL-6) in the serum samples were detected and compared using enzyme-linked immunosorbent assay (ELISA). The method was as follows: the serum samples and standard sample were placed in the reaction wells, incubated, washed down from the plate and patted to dry, added with working fluid and chromogenic agent, added with the elimination agent after the end of the reaction, and shaken to mix. The optic density (OD) of these samples was read on an enzyme micro-plate reader at a wavelength of 450 nm. Then, the standard curve was drawn according to the standard concentration. The cut-off index (COI) was used to represent the CP-IgA titer. When the COI was > 1.1, it was diagnosed as positive CP infection. The severity of coronary artery lesions in the case group was graded according to the results of the coronary angiography. The standards for scoring were as follows: the severity of stenosis of a single vessel of < 50% was set as 0.5 point, the severity of stenosis of a single vessel of ≥50% was set as 1 point, and the severity of stenosis of the left main trunk vessel of ≥50% was set as 3 points; a score within 0.5–1.5 was regarded as grade I, a score within 1.5–2.5 was regarded as grade II, and a score > 2.5 was regarded as grade III. The incidence of perioperative cardiovascular events, such as coronary dissection, coronary spasm, intracoronary thrombosis, no-reflow, acute myocardial infarction, re-interventional therapy or surgical bypass surgery, and cardiac death were observed and recorded in patients in the case group during the operation, or within one week after the operation. Patients in the case group were followed up for six months after the operation, and the follow up methods were telephone follow-up and follow-up visit. The incidence of adverse prognostic events, such as re-PCI, coronary artery bypass grafting (CABG) and cardiac death, were followed up and recorded.

### Statistical analysis

Data were statistically analyzed using statistical software SPSS 19.0. The serum CP-IgA, hs-CRP and IL-6 levels were expressed as mean ± standard deviation (x ± SD). Comparisons among groups were conducted using univariate analysis of variance. Pairwise comparison was conducted using the least significant difference (LSD). Variables were compared between the two groups using independent sample *t*-test. The non-normally distributed continuous data were compared using non-parametric tests. The CP infection rate was expressed in percentage (%), and evaluated using Chi-square test. *P* < 0.05 was considered statistically significant.

## Results

### Comparison of CP infection rate among patients with different severities of CHD

For patients in the case group, 39 patients were positive for CP-IgA and the CP infection rate was 42.4%. All subjects in the control group were negative for CP-IgA, and the CP infection rate was 0. The difference between these two groups was statistically significant (*X*^2^ = 29.221, *P* = 0.000). In the present study, among these 92 patients with CHD, 17 patients had grade I coronary artery disease, 39 patients had grade II coronary artery disease and 36 patients had grade III coronary artery disease, the CP infection rates were 17.6, 30.8 and 66.7%, respectively, and the difference was statistically significant (*X*^2^ = 15.106, *P* = 0.000). Among these patients, the difference in CP infection rate between patients with grade I coronary artery disease and patients with grade II coronary artery disease was not statistically significant (*X*^2^ = 1.040, *P* = 0.308). However, the CP infection rate was significantly higher in patients with grade III coronary artery disease than in patients with grade I coronary artery disease or grade II coronary artery disease, and the differences were statistically significant (*X*^2^ = 11.103, *P* = 0.000; *X*^2^ = 9.665, *P* = 0.002; Table 2).

**Table 2:** The Chlamydia pneumoniae among the patients with different grades of coronary artery lesions

Grade I coronary artery disease(*n* = 17)		Grade II coronary artery disease(*n* = 39)		Grade III coronary artery disease (*n* = 36)	
Infection cases	Infection rate	Infection cases	Infection rate	Infection cases	Infection rate
3	17.6^c^	12	30.8^c^	24	66.7^ab^
χ^2^	15.106				
P	0.000				

a.To compared with Grade I coronary artery disease, *P* < 0.05; b. To compared with Grade II coronary artery disease, *P* < 0.05; c. To compared with Grade III coronary artery disease, *P* < 0.05

### Comparison of serum CP antibody, hs-CRP and IL-6 levels in patients with different severities of CHD

The differences in serum CP-IgA, hs-CRP and IL-6 levels between patients with different severities of coronary artery disease and subjects in the control group were statistically significant (*P* = 0.000). With the aggravation of the severity of the coronary artery disease, the serum CP-IgA, hs-CRP and IL-6 levels gradually increased in patients with CHD, all were significantly higher, when compared to those in the control group, and the differences were statistically significant (*P* = 0.000, Table 3).

**Table 3:** The serum CP-IgA, hs-CRP, IL-6 levels among the patients with different grades of coronary artery lesions ($\overline{x}$ ±s)

Index	Grade I coronary artery disease (*n* = 17)	Grade II coronary artery disease(*n* = 39)	Grade III coronary artery disease (*n* = 36)	Control group (*n* = 50)	*F*	*P*
CP-IgA (COI)	0.62 ± 0.48^bcd^	1.01 ± 0.75^acd^	1.48 ± 0.82^abd^	0.33 ± 0.12^abc^	9.037	0.002
hs-CRP (mg/L)	12.55 ± 8.038^bcd^	28.67 ± 9.06 ^acd^	36.98 ± 13.13^abd^	7.46 ± 7.16^abc^	25.266	0.000
IL-6(ng/L)	1.17 ± 0.528^bcd^	2.15 ± 0.97 ^acd^	3.48 ± 1.24^abd^	0.52 ± 0.38^abc^	11.068	0.000

a.To compared with Grade I coronary artery disease, *P* < 0.05; b. To compared with Grade II coronary artery disease, *P* < 0.05; c. To compared with Grade III coronary artery disease, *P* < 0.05

d. To compared with control group, *P* < 0.05。

### Comparison of serum CP antibody, hs-CRP and IL-6 levels in CHD patients with different perioperative conditions

Perioperative cardiovascular events occurred in 21 patients, and the incidence was 22.8%. Among these patients, coronary artery dissection occurred in eight patients during the operation, coronary artery spasm occurred in seven patients during the operation, intracoronary thrombosis occurred in three patients, acute myocardial infarction occurred in two patients after the operation, and cardiac death occurred in one patient. Serum hs-CRP and IL-6 levels were significantly higher in patients with perioperative cardiovascular events, than in patients without perioperative cardiovascular events, and the differences were statistically significant (*P* = 0.000). However, the difference in serum CP-IgA level between these two types of patients was not statistically significant (*P* > 0.05, Table 4).

**Table 4:** The serum CP-IgA, hs-CRP, IL-6 levels between the patients with different conditions of perioperative cardiovascular events ($\overline{x}$ ±s)

Index	patients with perioperative cardiovascular events(*n* = 21)	patients without perioperative cardiovascular events(*n* = 71)	t	*P*
CP-IgA (COI)	1.38 ± 1.08	1.09 ± 0.82	1.320	0.105
hs-CRP (mg/L)	42.26 ± 22.36	24.07 ± 18.85	3.720	0.000
IL-6(ng/L)	3.61 ± 3.06	1.82 ± 2.23	2.955	0.002

### Comparison of serum CP antibody, hs-CRP and IL-6 levels in CHD patients with different prognoses

During the six-month follow-up after the operation, adverse prognostic events occurred in 11 patients, and the incidence was 12%. Furthermore, three patients were treated with CABG, while cardiac death occurred in two patients. In addition, serum CP-IgA levels were significantly higher in patients with adverse prognostic events than in patients without adverse prognostic events, and the differences were statistically significant (*P* = 0.000). Moreover, the differences in serum hs-CRP and IL-6 levels between these two types of patients were not statistically significant (*P* > 0.05, Table 5).

**Table 5:** The serum CP-IgA, hs-CRP, IL-6 levelsbetween the patients with different conditions of adverse prognosis events ($\overline{x}$ ±s)

Index	Adverse prognostic (*n* = 21)	Favourable prognosis(*n* = 71)	t	P
CP-IgA (COI)	1.78 ± 0.78	1.01 ± 0.73	4.181	0.000
hs-CRP (mg/L)	31.56 ± 16.28	29.51 ± 22.64	0.386	0.604
IL-6(ng/L)	2.56 ± 2.09	2.42 ± 2.72	0.217	0.781

## Discussion

The results of the present study revealed that patients with CHD have a higher CP infection rate, and the CP infection rate and serum levels of CP-IgA, hs-CRP and IL-6 also increased with the exacerbation of the severity of CHD. This suggests that CP infection and inflammation play important roles in promoting the pathogenesis and progression of CHD. Recently, the investigation on new risk factors for the occurrence and development of CHD has become a hot academic topic. The study on the role of pathogenic microorganism infection in CHD has attracted much attention on this background. CP can be propagated through peripheral blood mononuclear cells and replicated in vascular cells after entering the vascular tissue, and can promote the occurrence and persistence of inflammation through a variety of intracellular signaling pathways, such as the nuclear factor-κB signaling pathway. This affects the physiological functions of vascular endothelial cells, mononuclear-macrophages, smooth muscle cells and platelets, and induces oxidative stress, eventually causing damage to endothelial cells, the formation of foam cells and the proliferation of smooth muscle cells, and leading to the formation of coronary atherosclerosis. This is the basic mechanism of CP in the pathogenesis of CHD. The results of recent studies have revealed that [12, 13] CP infection is correlated to carotid intima-media thickness (IMT) in patients with CHD. Furthermore, the level of IMT is significantly higher in CHD patients with CP infection than in CHD patients without CP infection, and unstable plaques mostly manifest as common in carotid plaques. The results of the study conducted by Oh J et al. revealed that [14, 15] CP infection was also correlated to the severity of CHD, and that the positive rate of serum CP-IgG was significantly higher in patients with acute myocardial infarction and unstable angina pectoris, when compared to patients with stable angina pectoris or healthy people. In the study conducted by Yuguang Sun et al. [16–19], the researchers analyzed the correlation between CP infection and levels of inflammatory factors and blood lipids in patients with CHD. The results revealed that high CP infection rate, dyslipidemia and the overexpression of inflammatory factors, such as IL-6, hs-CRP and tumor necrosis factor-ɑ (TNF-α), can be commonly found in patients with CHD. Moreover, these pathological changes were highly correlated. Through clinical observation, all the above-mentioned study results confirmed that CP infection, dyslipidemia and inflammatory response may jointly play an important role in the pathogenesis of CHD and atherosclerosis, and as an important pathological mechanism in pathogenesis of CHD.

The present study revealed that the CP-IgA level was higher in patients with poor prognosis. This suggests that the prognosis of patients with CHD who underwent PCI has a certain correlation with the severity of CP infection. This may be because the formation of CP infection can continue to promote coronary arteriosclerosis plaque after PCI, and that this was also correlated to the damage to vascular endothelial function caused by CP infection-induced persistent inflammatory response. Sygitowicz G et al. revealed that [20, 21] CP infection and hyperlipidemia have a superposition effect on atherosclerosis, when compared to simple hyperlipidemia, and hyperlipidemia combined with CP infection can not only increase the expression of IL-1β and TNF-a, but also increase the area of atherosclerotic plaques. It was found that the plaque area of a CP-infected animal model increased by 25%, when compared with the control group, and that the plaque area of a hyperlipidemia animal model with CP infection was 23% larger than that of a simple hyperlipidemia animal model. This mechanism may be correlated to the ability of CP infection to decrease the protein expression of extracellular signal-regulating kinase (p-ERK1/2) and p-P38 by affecting the MAPK signaling pathway. A study also revealed that [22] CP infection could increase the levels of total cholesterol, free cholesterol and cholesteryl ester in THP-1 macrophage-derived foam cells, and decrease the level of cholesterol outflow. This reflects that CP can increase cholesterol levels in atherosclerotic plaques by promoting macrophage lipid accumulation and producing cholesterol outflow obstacles. Its mechanism may be correlated to the regulation of CP on the LXRα-ABCA1 signaling pathway. A study conducted by Min Zhou et al. revealed that [23] the levels of serum endothelin-1 (ET-1), fibrinogen (Fg), soluble vascular cell adhesion factor-1 (sVCAM-1), creatine kinase (CK), creatine kinase isoenzyme (CK-MB) and troponin I (CTn I) were higher in elderly CHD patients with CP infection, when compared to elderly CHD patients without CP infection. This shows that CP infection can not only increase the levels of inflammatory factors in patients with CHD, but also increase the damage to vascular endothelial function and myocardial function. This increases the risk of cardiovascular events in patients, and azithromycin treatment can effectively stabilize the patient’s condition. Wen Xiao et al. confirmed that [24] the CP-IgG positive rate was higher in patients with unstable angina pectoris, while the activities of tissue plasminogen activator (tPA) and plasma plasminogen activator inhibitor-1 (PAT-1) were higher in CP-IgG positive patients. This also shows that CP infection is closely correlated to relatively serious unstable angina pectoris. The above-mentioned studies suggest that CP infection can cause persistent arterial endothelial damage, such damage that may lead to the recurrence of cardiovascular events in CHD patients after PCI. This may be a mechanism underlying the correlation between CP infection and poor prognosis in patients with CHD. It is particularly noteworthy that the results of recent studies revealed that in addition to CP, the infections of other pathogenic microorganisms are also correlated to high levels of serum inflammatory factors in patients with CHD. Sfyroeras GS et al. revealed that [25] patients with CHD had a co-infection of CP with pathogenic microorganisms, such as *Helicobacter pylori* (Hp) and human cytomegalovirus (HCMV), and the long-term, repeated and chronic mixed infections of various pathogenic microorganisms could induce synergies between inflammatory markers, which jointly promote the occurrence and development of CHD. Therefore, CP infection may be one of the infectious factors that lead to poor prognosis in patients with CHD. The roles of various microbial infections, including CP infection, in the occurrence and outcome of CHD should be comprehensively evaluated, and should be the focus of further research. However, the prognosis of patients with CHD remains unknown and need further research.

## Conclusions

In summary, patients with CHD have higher CP infection rates, and the severity of infection is correlated with the severity of coronary artery disease and prognosis after PCI. This suggests that CP infection may be an important factor that affects the occurrence and outcome of CHD.

## Abbreviations

CABG: Coronary artery bypass grafting
CHD: Coronary heart disease
CK: Creatine kinase
CK-MB: Creatine kinase isoenzyme
COI: Cut-off index
CP: Chlamydia pneumoniae
CP-IgA: Chlamydia pneumoniae IgA antibody
CTn I: Troponin I
DSA: Digital subtraction angiography
ELISA: Enzyme-linked immunosorbent assay
ET-1: Endothelin-1
Fg: Fibrinogen
HCMV: Human cytomegalovirus
Hp: Helicobacter pylori
hs-CRP: Hypersensitive C-reactive protein
LSD: Least significant difference
OD: Optic density
PAT-1: Plasminogen activator inhibitor-1
PCI: Percutaneous coronary intervention
PTCA: Percutaneous transluminal coronary angioplasty
sVCAM-1: Soluble vascular cell adhesion factor-1
tPA: Tissue plasminogen activator

## References

[1] Wang EY, Dixson J, Schiller NB, Whooley MA (2017). Causes and predictors of death in patients with coronary heart disease (from the heart and soul study)[J]. Am J Cardiol.

[2] Huang J, Tang X, Ye F, He J, Kong X (2016). Clinical therapeutic effects of aspirin in combination with Fufang Danshen Diwan, a traditional Chinese medicine formula, on coronary heart disease: a systematic review and meta-analysis [J]. Cell Physiol Biochem.

[3] Sanchis-Gomar F, Perez-Quilis C, Leischik R, Lucia A (2016). Epidemiology of coronary heart disease and acute coronary syndrome [J]. Ann Transl Med.

[4] Gupta R, Mohan I, Narula J (2016). Trends in coronary heart disease epidemiology in India [J]. Ann Glob Health.

[5] Selwaness M, Bos D, van den Bouwhuijsen Q, Portegies ML, Ikram MA, Hofman A (2016). Carotid atherosclerotic plaque characteristics on magnetic resonance imaging relate with history of stroke and coronary heart disease [J]. Stroke.

[6] Hague WE, Simes J, Kirby A, Keech AC, White HD, Hunt D (2016). LIPID study investigators. Long-term effectiveness and safety of pravastatin in patients with coronary heart disease: sixteen years of follow-up of the LIPID study [J]. Circulation.

[7] Kianoush S, Al Rifai M, Cainzos-Achirica M, Umapathi P, Graham G, Blumenthal RS (2016). Update on the utility of coronary artery calcium scoring for coronary heart disease and cardiovascular disease risk prediction [J]. Curr Atheroscler Rep.

[8] Tang Y, Gao X, Shen J, Sun L, Yan W (2016). Epidemiological and clinical characteristics of Kawasaki disease and factors associated with coronary artery abnormalities in East China: nine years experience [J]. J Trop Pediatr.

[9] Bielicka A, Zielnik-Jurkiewicz B, Podsiadły E, Prochorec-Sobieszek M, Rogulska J4, Demkow U (2016). Role of chlamydia pneumoniae in the pathogenesis of hypertrophy and adenoid tissue inflammation in children [J]. Otolaryngol Pol.

[10] Sessa R, Di Pietro M, Filardo S, Bressan A, Mazzuti L, Serafino S (2017). Lack of association of chlamydia pneumoniae with cardiovascular diseases in virologically suppressed HIV patients [J]. New Microbiol.

[11] Grayston JT, Belland RJ, Byrne GI, Kuo CC, Schachter J, Stamm WE (2015). Infection with chlamydia pneumoniae as a cause of coronary heart disease: the hypothesis is still untested [J]. Pathog Dis.

[12] Rai NK, Choudhary R, Bhatia R, Singh MB, Tripathi M, Prasad K (2011). Et eal. Chlamydia pneumoniae seropositivity in adults with acute ischemic stroke: a case-control study [J]. Ann Indian Acad Neurol.

[13] Ionita CC, Siddiqui AH, Levy EI, Hopkins LN, Snyder KV, Gibbons KJ (2011). Acute ischemic stroke and infections [J]. J Stroke Cerebrovasc Dis.

[14] Oh J, Park S, Yu HT, Chang HJ, Lee SH, Kang SM (2016). Lack of superiority for soluble ST2 over high sensitive CReactive protein in predicting high risk coronary artery calcium score in a community cohort [J]. Yonsei Med J.

[15] Curb JD, Abbott RD, Rodriguez BL, Sakkinen P, Popper JS, Yano K (2003). C-reactive protein and the future risk of thromboembolic stroke in healthy men. Circulation.

[16] Shah CP, Shah BP, Dani SI, Channa BB, Lakshmanan SS, Krishnamani NC (2016). Efficacy and safety of the intensive dose of rosuvastatin 40mg/day in patients with acute coronary syndrome and at high risk of cardiovascular disease-ROSUVEES-2[J]. Indian Heart J.

[17] Jackson Lisa A., Smith Nicholas L., Heckbert Susan R., Grayston J. Thomas, Siscovick David S., Psaty Bruce M. (2000). Past Use of Erythromycin, Tetracycline, or Doxycycline Is Not Associated with Risk of First Myocardial Infarction. The Journal of Infectious Diseases.

[18] Dai D, Xiong W, Fan Q, Wang H, Chen Q, Shen W (2016). Association of decreased serum sTREM-1 level with the severity of coronary artery disease: inhibitory effect of sTREM-1 on TNF-alpha- and oxLDL-induced inflammatory reactions in endothelial cells [J]. Medicine (Baltimore).

[19] Yang A, Kang B, Choi SY, Cho JB, Kim YJ, Jeon TY (2015). Acute necrotizing pancreatitis associated with mycoplasma pneumoniae infection in a child [J]. Pediatr Gastroenterol Hepatol Nutr.

[20] Sygitowicz G, Tomaniak M, Filipiak KJ, Kołtowski Ł, Sitkiewicz D (2016). Galectin-3 in patients with acute heart failure: preliminary report on first polish experience [J]. Adv Clin Exp Med.

[21] Vallance P, Collier J, Bhagat K (2001). Infection, does acute endothelial dysfunction provide a link. Lancet.

[22] Elkind MSV, Carty CL, O’Meara ES, Lumley T, Lefkowitz D, Kronmal RA (2011). Hospitalization for infection and risk of acute ischemic stroke: the cardiovascular health study [J]. Stroke.

[23] Zhou M, Xu XW, Yang LJ, Kang AM, Liu SQ, Li YB (2014). Effect of chlamydia pneumoniae infection on disease progression in elderly patients with coronary heart disease [J]. Chinese Journal of Nosocomiology.

[24] Xiao W,Wang YQ,JiaXL,Xue HL, Wang JW (2015). Effect of chlamydia pneumoniae infection on the process of pathological changes of patients with acute cerebral infarction [J]. Chinese Journal of Nosocomiology.

[25] Sfyroeras GS, Roussas N, Saleptsis VG, Argyriou C, Giannoukas AD (2012). Association between periodontal disease and stroke [J]. J Vasc Surg.

